# Assessing the Impact of New Technologies on Managing Chronic Respiratory Diseases

**DOI:** 10.3390/jcm13226913

**Published:** 2024-11-16

**Authors:** Osvaldo Graña-Castro, Elena Izquierdo, Antonio Piñas-Mesa, Ernestina Menasalvas, Tomás Chivato-Pérez

**Affiliations:** 1 Departamento de Ciencias Médicas Básicas, Instituto de Medicina Molecular Aplicada (IMMA-Nemesio Díez), Facultad de Medicina, Universidad San Pablo-CEU, CEU Universities, 28925 Alcorcón, Spain; osvaldo.granacastro@ceu.es (O.G.-C.); elena.izquierdoalvarez@ceu.es (E.I.); 2 Departamento de Humanidades—Sección de Pensamiento Facultad de Humanidades y Ciencias de la Comunicación, Universidad San Pablo-CEU, CEU Universities, 28003 Madrid, Spain; anpime@ceu.es; 3 ETSI Informáticos, Centro de Tecnología Biomédica, Universidad Politécnica de Madrid, 28223 Pozuelo, Spain; ernestina.menasalvas@upm.es

**Keywords:** chronic respiratory diseases (CRDs), chronic obstructive pulmonary disease (COPD), asthma, artificial intelligence (AI), machine learning (ML), deep learning (DL), large language models (LLMs), remote patient monitoring (RPM), telehealth, ethical considerations in AI

## Abstract

Chronic respiratory diseases (CRDs), including asthma and chronic obstructive pulmonary disease (COPD), represent significant global health challenges, contributing to substantial morbidity and mortality. As the prevalence of CRDs continues to rise, particularly in low-income countries, there is a pressing need for more efficient and personalized approaches to diagnosis and treatment. This article explores the impact of emerging technologies, particularly artificial intelligence (AI), on the management of CRDs. AI applications, including machine learning (ML), deep learning (DL), and large language models (LLMs), are transforming the landscape of CRD care, enabling earlier diagnosis, personalized treatment, and enhanced remote patient monitoring. The integration of AI with telehealth and wearable technologies further supports proactive interventions and improved patient outcomes. However, challenges remain, including issues related to data quality, algorithmic bias, and ethical concerns such as patient privacy and AI transparency. This paper evaluates the effectiveness, accessibility, and ethical implications of AI-driven tools in CRD management, offering insights into their potential to shape the future of respiratory healthcare. The integration of AI and advanced technologies in managing CRDs like COPD and asthma holds substantial potential for enhancing early diagnosis, personalized treatment, and remote monitoring, though challenges remain regarding data quality, ethical considerations, and regulatory oversight.

## 1. Introduction

Chronic respiratory diseases (CRDs), such as asthma and chronic obstructive pulmonary disease (COPD), place a significant burden on healthcare systems. By 2050, the number of global COPD cases is projected to rise to 600 million, marking a 23% increase from 2020 [1]. They are both major global health issues [2]. Despite decreased age-standardized rates, they still cause significant morbidity and mortality, especially in low-income countries. Updated epidemiological trends are crucial for policy and healthcare training [3].

COPD is a common and progressive disease that primarily affects adults, particularly individuals over the age of 40 [4,5]. The prevalence increases with age, making it most common among those aged 60 years and older. Remarkably, COPD ranks as the third leading cause of death worldwide [6,7], a fact that has led the World Health Organization (WHO) to include COPD in the WHO Global Action Plan for the Prevention and Control of Noncommunicable Diseases (NCDs) and the United Nations 2030 Agenda for Sustainable Development.

The initial diagnosis is based on the clinical history and is confirmed by spirometry, which reveals airway obstruction. Spirometry also helps determine the severity of the disease, allowing for classification according to established guidelines for obstructive lung disease [8]. The information provided using new biomarkers, such as Surfactant Protein B Plasma Levels, and their possible role in the diagnosis and prediction of the progression of COPD have recently been investigated [9,10]. COPD exacerbations are acute episodes where respiratory symptoms worsen, usually one to four times a year, often due to infections or environmental pollution [11]. COPD is a chronic, progressive disease. While chest X-rays (CXRs) are useful for initial evaluations and during exacerbations, chest computed tomography (CT) scans provide more detailed imaging, identifying conditions like emphysema, bronchiectasis, or fibrosis [12].

Bronchial asthma is a common chronic inflammatory disease of the airways with a global prevalence ranging between 1 and 18%. It is characterized by reversible bronchial obstruction and the existence of bronchial hyperreactivity and is responsible for 250,000 deaths annually and represents a major cause of absenteeism from both work and school [13]. Diagnosis is based on clinical history, physical examination, and respiratory function tests [14]. In addition, airway inflammation can be easily assessed by quantifying the fraction of exhaled nitric oxide (FeNO) [15]. Usually, an allergy study is also necessary to check for possible sensitization to different aeroallergens [16]. Treatment varies according to the severity—mild, moderate, or severe—and whether the condition is intermittent or persistent, guided by both national and international clinical guidelines [14]. The assessment of static and dynamic lung volumes is crucial in diagnosing and monitoring various respiratory diseases, such as COPD and asthma [17]. Static lung volumes include measurements like the total lung capacity (TLC), residual volume (RV), functional residual capacity (FRC), and vital capacity (VC). These volumes provide information about the overall size of the lungs and any possible restrictions in lung expansion. CT and magnetic resonance imaging (MRI) can provide detailed anatomical measurements of lung volumes. Dynamic lung volumes focus on airflow rates during forced breathing measures, such as the forced vital capacity (FVC), forced expiratory volume in 1 s (FEV1), and the FEV1/FVC ratio. These measurements assess the ability of the lungs to move air quickly and are crucial in diagnosing obstructive lung diseases. Spirometry is the most widely used test to measure dynamic lung volumes and airflow.

Eosinophilia (increased levels of eosinophils in the blood or airways) plays a crucial role in evaluating asthma severity, predicting exacerbations, and guiding treatment, especially in patients who have features overlapping with COPD [18]. The effective management of COPD and asthma needs continuous monitoring, personalized treatments, and proactive interventions. Emerging technologies like artificial intelligence (AI), big data, sensors, and advanced imaging are revolutionizing CRD management. These innovations improve patient outcomes by enhancing patient–provider communication, supporting better diagnostic accuracy, enabling real-time monitoring, and facilitating personalized treatment approaches. Particularly, large language models (LLMs) streamline data management and improve clinical decision-making, paving the way for more efficient and precise care.

This article aims to evaluate the impact of new technologies on managing CRDs. It will analyze their effectiveness, accessibility, cost-effectiveness, and acceptability, while also exploring ethical implications like data privacy, bias, and artificial intelligence transparency. The goal is to provide insights into future respiratory care advancements.

## 2. State of the Art

**Figure 1:**
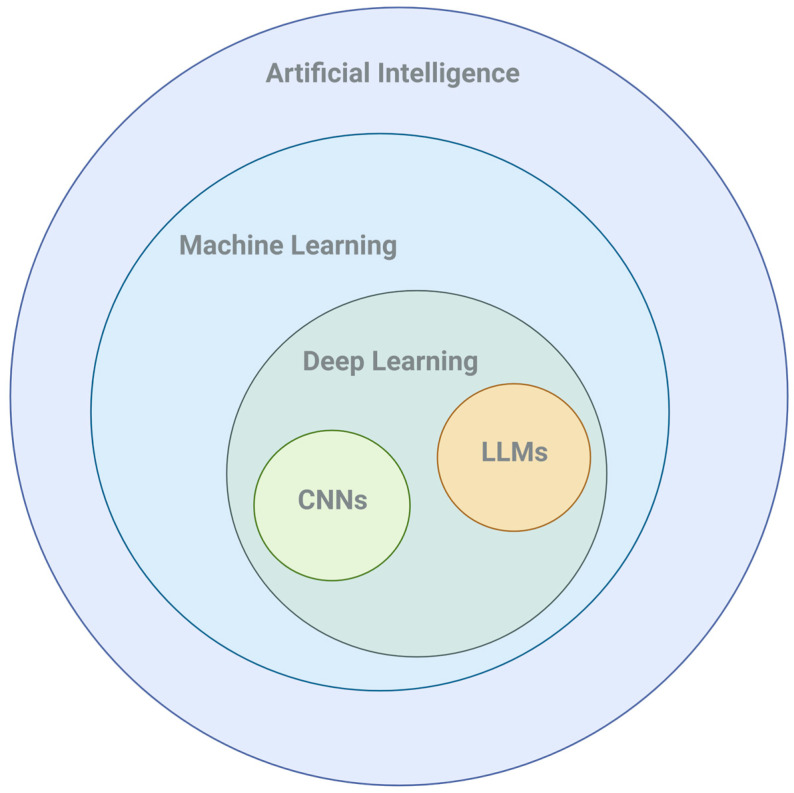
Layers of artificial intelligence. Created in BioRender.com.

AI is revolutionizing CRD management, enhancing early diagnosis, personalized treatment, and patient monitoring through machine learning (ML), deep learning (DL), and natural language processing technologies, like LLM (Figure 1, Table 1). This progress has significantly accelerated in recent years.

AI encompasses a broad range of technologies designed to replicate human intelligence. ML is a branch of AI and computer science that focuses on using data and algorithms to enable AI to imitate the way that humans learn, gradually improving its accuracy. ML has been defined as follows: “A computer program is said to learn from experience E with respect to some class of tasks T and performance measure P if its performance at tasks in T, as measured by P, improves with experience E” [19].

DL, a specialized form of ML, uses multi-layered neural networks to handle large volumes of complex data, excelling in tasks like image analysis and natural language processing. Neural networks in AI represent a model inspired by the structure and function of the human brain [20]. DL models, particularly convolutional neural networks (CNNs), excel in image analysis due to their capability to automatically learn spatial hierarchies of features from input images [21]. CNNs are tailored to process structured grid data, such as pixels in medical images, and extract key features like patterns or shapes, which are crucial for detecting abnormalities in lung scans associated with COPD [22,23]. Radiomics involves extracting numerous features from medical images using data-characterization algorithms [24]. These features, often imperceptible to the human eye, offer detailed insights into tissue characteristics such as shape, texture, and intensity. In the context of COPD, radiomics can analyze lung images, like CT scans or MRI, to detect subtle changes in lung tissue and structure that indicate disease progression. In fact, a multimodal approach incorporating CT imaging, demographic data, and spirometry has demonstrated superior ML predictions for COPD progression compared to using each modality individually [25]. The integration of CNNs into DL has revolutionized radiomics, enabling more precise and scalable analysis of medical images for COPD prognosis and management.

LLMs are DL models specifically trained on vast amounts of text data, making them highly effective in understanding and generating human language [26]. Increasingly, healthcare applications are integrating multimodal data, combining medical images, clinical data, and genetic information to improve the diagnostic and prognostic accuracy. Large Multimodal Models (LMMs) extend traditional LLMs by simultaneously processing these diverse data types, leveraging the full spectrum of patient information [27]. This approach can greatly enhance clinical decision-making by offering more precise predictions and tailored treatment plans for COPD patients, combining radiomic insights with other crucial health information.

**Figure 2:**
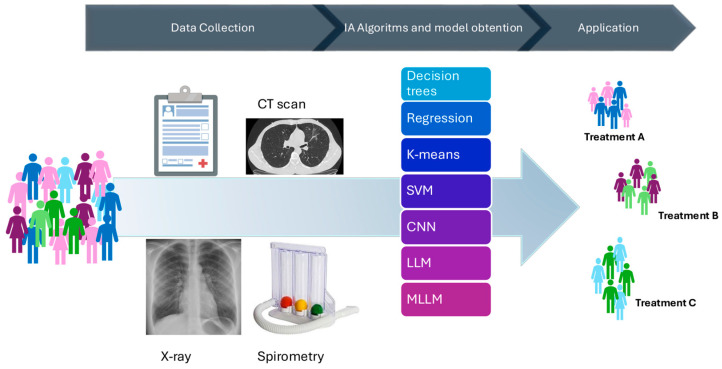
Data sources (medical records, X-ray, CT scans, and spirometry) and machine learning techniques (decision trees; regression models; k-means unsupervised clustering; SVM, support vector machine; CNN, convolutional neural network; LLM, large language model; MLLM, multimodal large language model) used to guide decisions for COPD treatment.

Despite the power of AI techniques such as ML, DL, LLMs, and CNNs, extracting meaningful insights from medical data remains a complex, multi-step process (Figure 2). It begins with disparate data sources, such as medical records, medical images, and spirometry results, which must be carefully curated, integrated, and quality-controlled. Essential data for applying AI in COPD includes demographic data, clinical measurements like FeNO, blood test results, and X-ray imaging data, among many others. These various types of data allow AI models to analyze biomarkers, inflammation levels, and lung structure, providing valuable information for accurate diagnosis, prognosis, and personalized treatment plans in COPD management.

Once this basic work is performed, various algorithms can be applied to uncover valuable patterns. For example, classification techniques such as support vector machines can identify different phenotypes in COPD patients based on lung function test patterns, while CNNs can analyze chest CT scans to detect early signs of emphysema. Throughout this process, General Data Protection Regulation compliance will be crucial to protect the privacy and security of patient data. In addition, results are only published and integrated into clinical practice after extensive validation and regulatory checks to ensure safety and efficacy in addressing medical problems.

## 3. Early Diagnosis and Prediction

The use of AI in the early diagnosis and prediction of CRDs is highly promising. ML algorithms can process vast amounts of patient records and clinical data to identify patterns often overlooked by traditional diagnostic methods [28]. Nowadays, ML algorithms are being more frequently incorporated into clinical practice for disease detection, diagnosis, and prognosis [29]. In the context of COPD, many healthcare professionals are leveraging ML to analyze COPD pathology and clinical diagnosis [30,31,32,33,34,35,36]. Most COPD-related studies use data from CT scans, genetic biomarkers, lung sounds, and pulmonary function tests. However, reproducibility remains a significant challenge due to limited access to large, standardized datasets and the high costs associated with these technologies.

Interestingly, the high-risk groups for COPD, the current screening methods, and the applications of AI in this field have been recently examined [37]. Different challenges such as data privacy, algorithm accuracy, and interpretability were pointed out. They suggest enhancing AI dissemination, improving data quality, fostering interdisciplinary cooperation, and securing policy and financial support to boost AI effectiveness in COPD screening in China. A recent study [38] has outlined an effective method for detecting respiratory diseases through audio recordings captured by electronic stethoscopes. The system employs a CNN and achieves high accuracy in classifying various respiratory conditions based on these audio signals. Additionally, a multimodal framework for identifying individuals sick with several chest diseases applying a CNN-based model by using CXRs, CT scans, and cough sound images (CSIs) has been recently shown [39]. This model uses techniques to deal with an imbalance of data and also ablation studies are presented to show the performance and robustness of the approach.

An in-depth analysis of the current state and future potential of AI in COPD diagnosis and management has explored AI methodologies but also emphasized the critical importance of clinical applicability in managing COPD [22]. It focuses on four main aspects: COPD identification and staging, emphysema subtyping, airway analysis, and the evaluation of vascular changes. This work highlights that AI-assisted diagnostics can improve accuracy and efficiency over traditional methods, potentially reducing misdiagnosis and enabling earlier COPD detection. Specifically, AI tools based on CT image analysis could significantly cut down the time radiologists and pulmonologists spend on interpreting images. Although the review acknowledges promising results in AI for COPD management, it also remarks on significant challenges such as small sample sizes, a lack of long-term data, and insufficient labeled examples. Thus, future research focusing on large-scale, diverse studies with extended follow-up periods is still needed. Additionally, this stresses the importance of developing interpretable AI models and integrating them into clinical workflows to build trust and confidence in AI-assisted decision-making.

The use of ML and DL for COPD management has been compared [40]. Both showed potential in improving mortality predictions over traditional regression models, but not in predicting exacerbations. Challenges include high variability, poor handling of missing data, small sample sizes, and a lack of confidence intervals, leading to bias. The study suggested integrating imaging with clinical data and prioritizing robust model development, 3D analysis, and multimodal data integration for better outcomes.

Regarding asthma management, over the past two decades, various technologies have been explored. Early studies focused on expert systems, artificial neural networks (ANNs), and ML to assist clinicians with diagnosing asthma, interpreting spirometry data, and classifying respiratory sounds [41,42,43,44,45,46,47,48]. While these early models showed promise, many remained in the experimental stages. More recent studies have expanded into telemonitoring, wearable devices, and AI-based decision support tools [49,50,51]. Studies have demonstrated ML’s potential in predicting asthma exacerbations and hospital readmissions [52,53]. AI-assisted tools, such as smart stethoscopes, have improved exacerbation detection [54]. Other innovations, like AI-based pulmonary function test interpretation [55] and ML for respiratory sound classification have enhanced the diagnostic accuracy [50]. Several of these methods, such as DL models for predicting readmissions and AI-assisted imaging for bronchiectasis, have demonstrated high accuracy. However, they still need additional validation [56]. Although many of these tools are still in development or undergoing clinical trials, smart inhalers, telemedicine platforms, and AI-assisted spirometry interpretation are already being implemented in real-world settings [53,54]. However, full integration into routine clinical practice remains limited due to challenges in standardization and regulatory approval.

## 4. Remote Monitoring and Telehealth Integration

Telehealth encompasses healthcare services delivered through audio and video technology. It was initially created to offer basic care to patients in rural and underserved areas [57]. Remote patient monitoring (RPM) involves collecting, transmitting, and evaluating patient health data through electronic tools such as wearable devices, mobile gadgets, smartphone apps, and internet-connected computers. RPM, also known as telemonitoring, is a telemedicine strategy that enables clinicians to remotely observe patients’ health parameters and intervene when abnormalities are detected [58]. RPM is crucial for COPD care [59]. Key parameters that are considered for telemedicine and RPM in COPD patients are related to vital signs (oxygen saturation, heart rate, blood pressure, and body temperature); respiratory parameters (respiratory rate, peak expiratory flow (PEF), spirometry, and sputum color and volume); symptom and health status tracking (daily questionaries, exacerbation symptoms, and medication usage); and physical activity and weight monitoring (activity levels and body weight) [60]. The integration of AI and RPM enables real-time patient observation, facilitating proactive care and early intervention [61]. AI plays a crucial role by analyzing large datasets, identifying trends, predicting health outcomes, and aiding decision-making, thereby enhancing the efficiency and effectiveness of remote care. Telemonitoring has been utilized for patients with COPD [62], and it enhances patient health by reducing blood pressure, decreasing the need for antibiotics and steroids, and minimizing the number of clinical consultations [63]. COPD telemonitoring even has the potential to predict exacerbations before they occur [64]. The integration of AI with telehealth has significantly improved the management of chronic respiratory conditions. AI-powered wearables monitor vital signs and respiratory metrics in real time, alerting healthcare providers to issues such as declining lung function for timely interventions. Additionally, AI enhances patient–provider communication, enabling virtual consultations and remote care, particularly benefiting underserved areas. Respiratory sensors [65,66] and pulse oximetry devices [67] detect symptoms remotely, aiding proactive management. A study [68] using ML (a decision tree forest) achieved 75.8% accuracy in predicting COPD exacerbations, highlighting the potential of AI in monitoring and managing patient health. Another study explored the role of AI in improving respiratory care [69]. This highlights AI’s potential to enhance diagnosis, treatment planning, and patient monitoring in conditions such as asthma, COPD, and other respiratory diseases. The article discusses AI’s ability to provide personalized treatment plans, real-time patient monitoring using wearable devices, and early disease detection through advanced algorithms. Additionally, it addresses challenges, such as data privacy, algorithmic bias, and the need for ethical standards, while also emphasizing future opportunities for AI integration in healthcare.

Telehealth has been used for asthma patients, relying on regular lung function tests like spirometry for diagnosis and monitoring [70]. An initial diagnosis requires in-person visits for tests such as bronchodilator response or FeNO measurement. However, ongoing monitoring can be managed through telehealth with proper patient training for at-home PEF measurement. Telehealth also helps triage patients who need further clinical evaluation if symptoms worsen. An initial study demonstrated that specialized care delivered via telehealth improved asthma symptoms and quality of life in pediatric patients [71]. Tools like short message services (SMSs), internet-based platforms, and real-time telehealth, when combined with patient education, can improve asthma outcomes, achieving results comparable to those of traditional in-person consultations [70].

## 5. The Role of Large Language Models

LLMs are sophisticated AI systems that are trained on extensive text datasets to comprehend, produce, and analyze human language [26]. LLMs aid clinicians by processing medical records, offering evidence-based recommendations, and synthesizing medical knowledge to improve decision-making. LLMs excel in processing and generating human-like text, revolutionizing patient education, treatment adherence, and clinical documentation. They create personalized educational content, simplify complex medical information, and help healthcare providers maintain accurate records. When combined with multimodal data—such as imaging, lab results, and clinical history—LLMs enhance diagnosis, prognosis, and personalized care plans. Their use in managing chronic respiratory diseases highlights the evolving role of AI in healthcare, improving precision and patient-centered outcomes. In COPD management, they analyze patient data, provide real-time insights from the literature, and could help personalize treatment plans [40].

A study has evaluated models such as chat generative pre-trained transformer (ChatGPT), GPT-4, and large language model Meta AI (LLaMA) in identifying patients with specific conditions using gold-labeled electronic health records (EHRs) from the MIMIC-III database [72]. The research covered three common diseases: COPD, chronic kidney disease, and the rare condition of primary biliary cirrhosis, as well as the challenging-to-diagnose condition, cancer cachexia. The findings underscore both the potential and the current limitations of LLMs in healthcare. While they showed promise in assisting clinicians with tasks like patient identification, the study also pointed out significant issues, including error rates, a lack of explanation, and ethical challenges such as data privacy and model transparency. These concerns suggest that LLMs, for now, would serve best as supplementary tools in clinical environments. Before they can be reliably integrated into real-world healthcare settings, improvements are needed to reduce false positives and false negatives.

## 6. Challenges and Ethical Considerations

From an ethical perspective, we observe that the application of AI has a positive effect by improving the quality of life of patients. The possibility of individualization will result in the effectiveness of the therapeutic relationship. But the important advances in the application of AI in CRDs are not without difficulties. Issues such as the data quality, potential algorithmic bias, and interpretability of AI models remain concerns. In addition, the integration of AI into clinical practice raises ethical issues related to patient privacy, informed consent, and the transparency of AI-based decisions. Addressing these challenges is essential to ensure that AI technologies are used responsibly and effectively in the treatment of chronic respiratory diseases.

The European AI regulation (EUR-Lex Document 32024R1689 Regulation (EU) 2024/1689 of the European Parliament [73]) is the first-ever legal framework on AI, which addresses the risks of AI. The Regulatory Framework defines four levels of risk for AI systems: unacceptable risk, high risk, limited risk, and minimal risk. None of the new technologies for managing chronic respiratory diseases are considered unacceptable or high-risk.

Of the technologies cited in this article, only chatbots would be included as limited-risk technologies. Limited risk refers to the risks associated with a lack of transparency in AI usage. The European regulation warns that when using AI systems such as chatbots, humans should be made aware that they are interacting with a machine so they can take an informed decision to continue or step back.

The new Code of Ethics of the General Council of Medical Associations of Spain [74] includes three articles that prevent the inappropriate use of AI and health databases. The Code reminds physicians that they must demand ethical and purposeful control of research with artificial intelligence based on the transparency, reversibility, and traceability of the processes in which they intervene, to guarantee patient safety (Chapter XXIV, p. 85). It also warns about respect for confidentiality (86.1) and the risk of the intentional manipulation of data or results obtained from large health databases (86.2). The risk of bias should not be attributed to the AI; it is the input data that is biased, so human supervision is necessary to verify the data and the results [75]. The priority should be to promote education and awareness of biases among IA developers and users [76].

Healthcare providers are becoming data interpreters, balancing AI-driven insights with clinical expertise to support informed decisions. Providers are also ethics guardians, ensuring AI tools are used responsibly and transparently. As system integrators, they work to seamlessly incorporate AI into clinical workflows, and as telehealth facilitators, they manage patient care remotely with AI-enabled tools. Lastly, they are committed to continuous learning, adapting to AI advancements and acquiring data science skills to better utilize these technologies in patient care.

## 7. Conclusions

The integration of AI and new technologies in the management of CRDs offers significant potential to enhance early diagnosis, personalized treatment, and remote patient monitoring. AI, particularly through ML, DL, and LLMs, has demonstrated efficacy in diagnosing diseases such as COPD and asthma, improving the accuracy and timeliness of interventions, and reducing the burden on healthcare providers. Moreover, telehealth and remote patient monitoring are reshaping patient care by providing real-time data, enabling proactive management, and improving patient outcomes, especially in underserved areas.

However, the implementation of these technologies is not without challenges. AI models face issues related to data quality, interpretability, and algorithmic bias. Ethical concerns, such as data privacy, transparency, and patient autonomy, must also be addressed to ensure the responsible use of AI in clinical practice. Despite these challenges, the future of CRD management looks promising, with AI-driven tools paving the way for more precise, efficient, and patient-centered healthcare. Moving forward, collaboration between healthcare professionals, AI developers, and policymakers will be crucial to overcome the obstacles and fully realize the benefits of these technologies in respiratory care.

## 8. Novelty of the Research

The novelty lies in our comprehensive examination of how AI techniques such as ML, DL, LLMs, and telehealth systems are transforming the diagnosis, treatment, and monitoring of CRDs. Key contributions include the following:

1. Integration of multimodal AI: The combination of different data types—such as medical imaging, spirometry, and clinical records—enhances diagnostic precision and enables more personalized treatments. LMMs that utilize both structured (e.g., images) and unstructured data (e.g., text) are particularly innovative, paving the way for a more holistic approach to patient management.
2. Early diagnosis and prediction with AI: AI models, especially CNNs and radiomics, improve the accuracy of early disease detection, and progression monitoring is a significant advancement. The use of audio analysis from electronic stethoscopes and multimodal frameworks (e.g., combining CXRs, CT scans, and clinical data) represents a novel approach to enhancing early diagnosis.
3. Remote monitoring and telehealth: The integration of AI with RPM demonstrates the potential for real-time, proactive healthcare. The application of wearable devices, telemonitoring platforms, and smart inhalers for managing COPD and asthma offers a glimpse into future healthcare models that prioritize accessibility and preventive care.
4. Ethical and regulatory insights: Our attention to ethical considerations and the implications of the European AI regulation provides a timely perspective on navigating the challenges of AI integration in healthcare, such as data privacy, bias, and transparency. This is crucial for guiding future developments in AI-powered healthcare tools.

## 9. Future Prospects and Potential Impact

Our research indicates a promising future for AI-driven healthcare, particularly in CRD management. Some potential impacts and directions for future exploration include the following:

1. Widespread adoption of AI in clinical practice: The successful integration of AI technologies into routine clinical workflows can reduce the burden on healthcare providers, improve diagnostic accuracy, and enhance patient outcomes. However, achieving this requires overcoming current barriers related to data standardization, model interpretability, and regulatory compliance.
2. Scalability and accessibility: The deployment of telehealth platforms and AI-powered remote monitoring tools can significantly enhance access to care, especially in underserved and rural areas. This could help bridge gaps in healthcare access, reduce hospital admissions, and facilitate continuous patient management outside traditional clinical settings.
3. Personalized medicine: By leveraging multimodal AI models that integrate genetic, imaging, and clinical data, there is potential for developing highly personalized treatment plans that optimize outcomes for individual patients. This could lead to more targeted therapies for CRDs, reducing the trial-and-error approach currently used in treatment.
4. Ethical AI in healthcare: The emphasis on ethical frameworks and regulatory guidelines highlights the need for responsible AI implementation. As these technologies become more embedded in healthcare systems, ensuring fairness, transparency, and patient autonomy will be critical. Future research should focus on developing explainable AI models that clinicians and patients can trust.
5. Addressing data limitations: We note challenges in accessing large, standardized datasets, which limit the reproducibility of AI models. Future initiatives could include collaborative data-sharing platforms that enhance data availability while safeguarding patient privacy.

Overall, the integration of AI into the management of chronic respiratory diseases has the potential to transform healthcare, making it more efficient, proactive, and patient-centered. However, realizing these benefits will require collaborative efforts among researchers, clinicians, policymakers, and technologists to address existing challenges and build robust, scalable solutions.

## Figures and Tables

**Table 1:** Summary of the key aspects discussed in the state of the art section.

Key Aspect	Description
AI in CRD Management	AI, through ML, DL, and NLP technologies (e.g., LLM), is enhancing early diagnosis, personalized treatment, and patient monitoring for CRD.
Machine Learning	ML uses data and algorithms to mimic human learning, improving accuracy over time. It is defined as a computer program that learns from experience (E) with a class of tasks (T) and performance measures (P) to improve its performance.
Deep Learning	A subset of ML that employs multi-layered neural networks to process large, complex data. DL excels in image analysis (e.g., CNNs for medical imaging) and NLP. CNNs automatically learn spatial hierarchies from structured grid data, which is valuable for detecting abnormalities in medical images.
Radiomics in COPD	Radiomics extracts numerous features from medical images (e.g., CT or MRI) using data-characterization algorithms. It provides insights into tissue characteristics that aid in COPD diagnosis and progression monitoring. DL models, particularly CNNs, enhance radiomics by enabling scalable analysis of medical images.
Large Language Models	LLMs, a type of DL model, excel in understanding and generating human language. They are increasingly used in healthcare to improve diagnostic accuracy and treatment planning, especially through multimodal integration with other patient data sources.
Large Multimodal Models	LMMs extend LLMs by processing diverse data types (e.g., medical images and clinical data) to enhance diagnostic accuracy and clinical decision-making, offering more precise predictions and treatment options for diseases like COPD.
Data Integration Process	Involves curation, integration, and quality control of various data sources (e.g., medical records, images, spirometry). Algorithms like CNNs and support vector machines are used for phenotype classification and image analysis. Data handling complies with GDPR for patient privacy and security. Extensive validation is required before clinical implementation.
